# Gaps in Population Life Course Phenotype Trajectories Underlying Major Noncommunicable Diseases: A Scoping Review

**DOI:** 10.23889/ijpds.v6i1.3346

**Published:** 2026-08-20

**Authors:** Katie McBain, Samuel Kasjan, Ryan Taylor, Dorothea Dumuid, Susan Clifford, Timothy Olds, Melissa Wake

**Affiliations:** 1 Murdoch Children’s Research Institute, Parkville, Victoria, Australia; 2 Faculty of Medicine, Dentistry & Health Sciences, The University of Melbourne, Parkville, Victoria, Australia; 3 Alliance for Research in Exercise, Nutrition and Activity (ARENA), Allied Health & Human Performance, University of South Australia, Adelaide, South Australia, Australia

**Keywords:** noncommunicable diseases, epidemiology, longitudinal studies, trajectory, life course

## Abstract

**Introduction:**

The life course phenotypic pathways leading to noncommunicable diseases (NCDs) provide information needed to plan and test preventive interventions. However, most NCD-relevant phenotypes are not routinely measured until diagnosis and their pre-clinical trajectories are therefore not in linked population datasets.

**Objectives:**

In the context of planning phenotypic collection waves in an Australian early and pre-midlife mega-cohort, we aimed to undertake (1) a scoping review to identify knowledge availability and gaps and (2) a comparative map of trajectories from available data.

**Methods:**

We searched PubMed and MEDLINE (September 2024) for trajectory studies on phenotypes underlying NCDs with the highest late life disease burden (excluding cancer and back pain, with no clear precursor phenotypes): cardiovascular, chronic obstructive pulmonary and kidney diseases, diabetes, falls, hearing and vision loss, and dementia. Eligible studies had ≥3 timepoints spanning ≥5 years in childhood or ≥10 years in adulthood. Using the R ggplot package, we fitted loess curves to create lifetime trajectory visualisations in absolute values and units standardised for comparison.

**Results:**

From 3770 abstracts, we included 36 studies. Most (n == 19) examined cardiovascular trajectories, collectively spanning ages 5–105 years for blood pressure. Ten studies reported cognition trajectories, but could not be synthesised due to measurement diversity. Twelve studies mapped lung, glucose, kidney or musculoskeletal phenotypes but with discontinuities at varying life stages. No studies tracked vision or hearing trajectories. Our syntheses confirmed some known trajectory patterns, such as peaking of musculoskeletal phenotypes in early adulthood and the rise in cardiovascular and glucose markers beyond healthy ranges from midlife.

**Conclusions:**

Our mapping confirmed expected patterns for some phenotypes, but highlighted significant gaps for others on pathways to high-burden NCDs. If long-running population cohorts collectively tracked all major phenotypes over time, embedded real-world or simulated trials could accelerate progress in prevention and treatment across all major NCDs.

## Introduction

If noncommunicable diseases (NCDs) could be prevented, excess population morbidity and early mortality could be curbed. In Australia and other high-income countries, NCDs cause approximately 90% of deaths [1]. Increases in life expectancy have come at the expense of quality of life, with rising multimorbidity and health costs [2]. While not the only answer, research is not keeping pace with need: more people are dying from diseases that should be preventable, life expectancy has stalled or reversed in many countries and healthcare costs continue to rise [3–6]. Many NCDs are shaped by lifelong health trajectories: how well we develop and how well we age [7]. Intervention is possible at any and all ages, but we still do not know the best timing, approach, target groups or effect sizes – whether for single diseases or multimorbidity.

Both unique and shared (such as chronic inflammation, stress) drivers shape the underlying trajectories to NCDs [8]. These trajectories can be seen as repeated phenotypic signals that track directly to our future health [9]. In this review, we define precursor, or intermediate, phenotypes as measurable, quantitative traits on the direct structural or functional pathways between genetic variation and disease [10, 11]. For example, we preference lung function as an intermediate phenotype (precursor to chronic obstructive pulmonary disease) over smoking (a behavioural risk factor), and carotid intima-media thickness over lipids as a precursor of large artery disease. Understanding lifelong trajectory patterns of phenotypes could inform preventive strategies by enabling “what-if” or real-world interventions and for estimating their impacts on trajectories both immediately and in the longer term, beyond the timeframes of a funded randomised trial. These preventive strategies may aim to maximise the peak functioning achieved during growth, extend the plateau at peak function, or slow the inevitable rate of decline with ageing [12, 13].

NCD-relevant phenotypes are not typically routinely measured in clinical settings until disease emerges and therefore do not appear in linked services datasets, especially during childhood and earlier adulthood. For many phenotypes, long-running cohort studies are the only sources of population-level longitudinal measurements [7]. While whole-population data may be assumed to equate to administrative, geographic or census data, technology now also enables the direct collection of phenotypic data at this scale. Multi-phenotypic, longitudinal population platforms offer the potential to map phenotype trajectories and test prevention strategies at different life stages across many diseases. One such study, Generation Victoria (GenV) [14] – Australia’s largest and most inclusive interventional early and pre-midlife cohort – provides an opportunity to consider this need at population scale. To date, GenV comprises over 120,000 Australians (nearly 45,000 children born between 2021–2023 and their parents) from all demographics and regions of Victoria (population 7 million in an area similar in size to the UK) [15]. GenV plans to conduct 5-yearly phenotypic data collection waves from early school age and pre-midlife.

To plan GenV’s phenotypic waves, we needed to review existing knowledge on NCD-relevant phenotype trajectories. We suspected that, although repeat measures of some phenotypes would be available from longitudinal cohort studies, they would rarely cover the entire life course, for instance spanning only parts of childhood or older adulthood [16]. The aims of this paper were therefore to (1) conduct a scoping review to identify knowledge availability and gaps in whole-of-life phenotypic trajectories to major NCDs in high-income countries at population-level and (2) comparatively map trajectories from available data.

## Methods

We undertook a scoping review following the JBI methodologi­cal guidance [17] and Preferred Reporting Items for Systematic Review and Meta-Analyses – Extension for Scoping Reviews (PRISMA-ScR) [18] reporting guidelines (checklist available in Supplementary material S1). We chose to conduct a scoping review to best address our study’s aims as, unlike systematic reviews, scoping reviews are used to map evidence and identify gaps [19]. We prespecified our aims and methodology in our protocol at [https://osf.io/29nsm]. Drawing on the papers identified with relevant trajectories, we then synthesised trajectories across studies, in both absolute and relative units, using an approach suited to the heterogeneity of available published sources.

**Table 1:** Phenotypes Considered in Scoping Review

**NCD**	**Intermediate phenotype(s)**
Cardiovascular diseases (ischaemic heart disease, stroke, hypertensive heart disease)	Vascular function and stiffness Blood pressure (systolic and diastolic) Augmentation index Pulse wave velocity Retinal microvasculature vessel calibre Carotid artery intima-media thickness, distensibility, elasticity
	Autonomic control Heart rate
Diabetes mellitus	Blood glucose Fasting blood glucose levels Haemoglobin A1c
Chronic obstructive pulmonary disease	Lung function Forced expiratory volume (FEV_1_) Forced vital capacity (FVC)
Adverse impacts of falls	Bone density and morphology Density Geometry Size Strength
	Muscle strength and morphology Strength (e.g., grip strength) Mass
Chronic kidney disease	Albuminuria
	Estimated glomerular filtration rate
Age-related hearing loss	Hearing acuity
Blindness and vision loss	Visual acuity
Alzheimer’s Disease	Cognition Global cognition Verbal intelligence Performance-based intelligence Executive functioning Episodic memory

## Search Strategy

We searched Ovid MEDLINE and PubMed from inception until 17^th^ September 2024. The protocol also noted including articles from Embase, but initial review of a subset of articles yielded far fewer relevant articles so did not proceed. Our search strategy included search terms related to our NCDs and phenotypes of interest (see ‘*Concept*’ below), along with terms related to trajectories and longitudinal studies. Searches were limited to articles published in English. The full search strategy for Ovid MEDLINE is presented in Supplementary material S2.

## Eligibility Criteria

### Population

We included publications that reported phenotype trajectories where measurements were drawn from the general population and excluded those that reported on clinical samples.

### Concept

We first identified the top ten causes of global noncommuni­cable disease burden in adults aged 75 years and above from the Global Burden of Disease Study 2019 [20]. These were: cardiovascular diseases (ischaemic heart disease, stroke, hypertensive heart disease), chronic obstructive pulmonary disease, diabetes mellitus, falls, chronic kidney disease, hearing loss, blindness and vision loss, and Alzheimer’s disease. We included falls (technically an injury rather than an NCD) because there is evidence for the roles of muscle and bone phenotypes influencing the likelihood of falling and resulting in fracture [21, 22].

Although a top contributor to global disease burden, we excluded cancer because clear phenotypic trajectories leading to cancer are not evident and cancer has a large stochastic component [23]. We also excluded low back pain as evidence for its underlying phenotypic trajectories is limited and, unlike many other NCDs, it is usually episodic rather than progressive [24, 25].

Table 1 shows the list of phenotypes considered. This list is intended to be thorough but not exhaustive. To contain the search, we made pragmatic choices to include phenotypes that appear to be the most direct, population-measurable precursors of the highest-burden NCDs. Although a significant risk factor for many NCDs, we excluded trajectories of body composition because there is debate surrounding its status as a risk factor, risk marker or disease [26].

Trajectories can be constructed in various ways, but conceptually fall into three main categories: normative, group-based and endpoint-based. Normative trajectories show the typical pattern of a phenotype within a population [27]. Group-based trajectories group individuals into one of usually several patterns using group-based modelling strategies [28]. Lastly, endpoint-based trajectories are constructed retrospectively from disease endpoint and compare pathways in those diagnosed to healthy controls [29]. We included publications reporting normative population trajectories to characterise within-individual change relevant to population-level measurement and prevention. We excluded group-based and endpoint-based trajectories, as these do not yield a single, generalisable trajectory suitable for informing universal phenomic measurement or trial outcome design.

We included publications where trajectories were developed from a minimum of three measurement timepoints and spanning at least five years in childhood (age 0-20 years) [30] or ten years in adulthood (age 21 years and older). We chose these time periods to enable inferences to be drawn about patterns of change in phenotypes across the life course, recognising that rapid growth occurs inchildhood.

### Context

Because this work was intended to guide planning for an Australian mega-cohort, we included publications where trajectories were modelled in high-income countries (as defined by the World Bank) [31]. This is because of the notable differences in NCD aetiology, progression and life expectancy between high- and low- and middle-income countries (LMICs) [32, 33].

### Sources

We included publications where trajectories were developed from empirical data and excluded theoretical trajectories. We included trajectories modelled from longitudinal data and excluded those developed entirely from cross-sectional data. This is a deviation from our protocol [https://osf.io/29nsm] due to scope change to only focus on trajectories modelled from longitudinal data, because they are less impacted by cohort effects and other biases [34].

## Screening and Study Selection

We imported articles to Endnote [35] and removed duplicates using The Systematic Review Accelerator’s [36] Deduplicator tool, before importing articles to Covidence [37]. Two reviewers (KM and SK or RT) dual-screened articles by title and abstract and then reviewed the full texts of selected citations.

## Data Extraction

Both KM and SK extracted data from five randomly selected articles using a study-developed data extraction form in Covidence [37]. Once consensus of the extracted data was established, the remaining articles were extracted by either KM or SK individually. A risk of bias assessment was not undertaken as the purpose of conducting a scoping review is to map evidence rather than assess study quality.

## Trajectory mapping

### Individual Phenotype Synthesis

To synthesise trajectories of individual phenotypes, KM and DD used the ggplot2 [38] package in R, version 4.3.0 [39]. Where papers reported the mean phenotype values at each measurement timepoint, these were used in our synthesis. If these values were not reported, KM used Plot Digitizer [40] to extract values from figures at approximately yearly intervals. We used the ‘geom smooth’ function in ggplot2 to fit a loess curve through the datapoints from each paper, leaving gaps at ages where there was no coverage from the included studies. Where studies modelled trajectories spanning the same age ranges in different birth cohorts (e.g., to investigate secular trends) we included only the most recent birth cohort to minimise cohort effects. Where studies separated trajectories by sex, we synthesised separate male and female trajectories in addition to an overall trajectory.

### Comparative Synthesis

To place individual phenotype trajectories on the same scale to enable comparison, we converted values to a percentage, representing a deviation from a healthy reference value. This healthy reference value was set to an obvious peak (e.g., grip strength) if available from our individual synthesised trajectories, or, if not available, selected from relevant literature or guidelines (see Supplementary material S3 for values). Where eligible studies reported trajectories separately for population sub-groups (e.g., sex) we analysed each sub-group separately and applied subgroup-specific reference or peak values (such as for muscle mass). These conversions allowed us to bring together the multiple phenotypes, each measured in different metrics, onto the same y-axis. After converting the values to percentage deviations, we again used the ggplot2 package in R 4.3.0 to fit loess curves.

## Results

### Literature search

**Figure 1:**
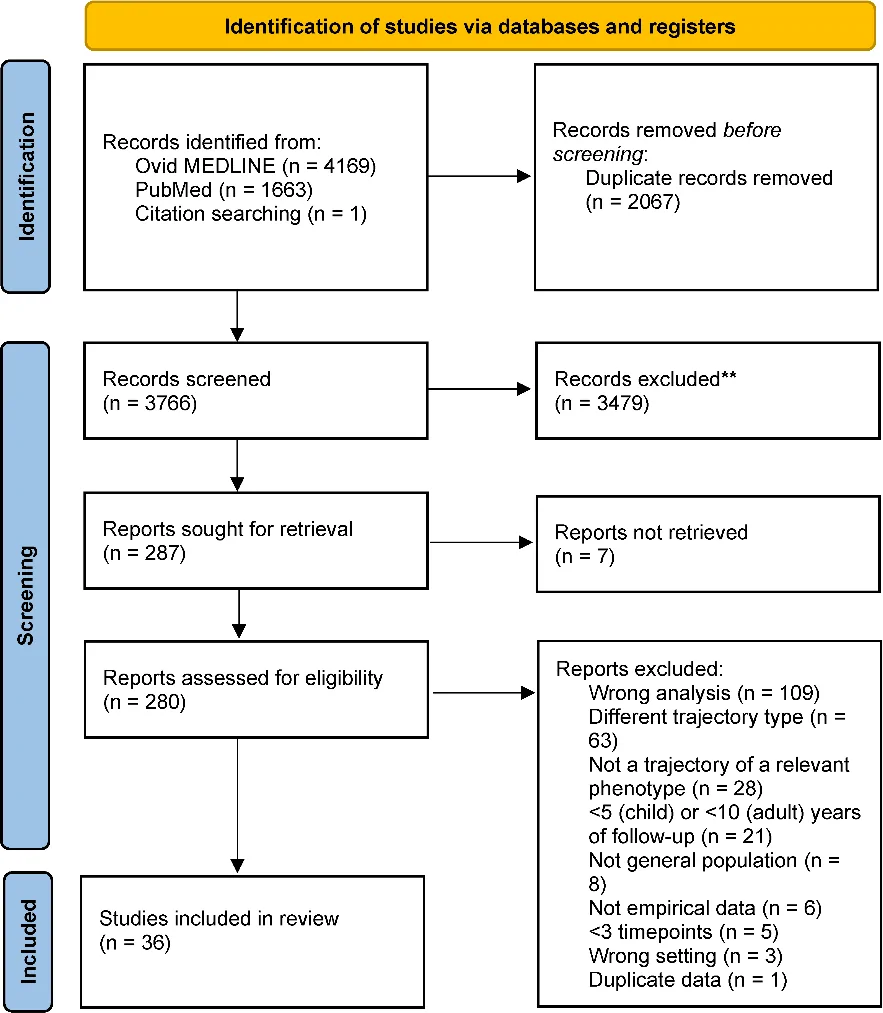
Figure 1: PRISMA Flow Chart

Source: Page MJ, et al. BMJ 2021;372:n71. https://doi.org/10.1136/bmj.n71.

We identified 5,833 records from databases and citation searches. After removing 2063 duplicate records, we reviewed 3,770 abstracts and 280 full texts, resulting in 36 articles included in this review [27, 34, 41–73]. Figure 1 summarises the study screening and selection process.

This work is licensed under CC BY 4.0. To view a copy of this license, visit https://creativecommons.org/licenses/by/4.0/.

### Study Characteristics

Table 2 shows the characteristics of the included studies. Most studies were conducted in the United States of America (USA, n = 17). Eight were conducted in the United Kingdom (UK), two each in Italy, Germany and the Netherlands, and one in each of Australia, Japan, Austria and Sweden. One study was international, spanning several European countries. Sample sizes ranged from 368 to 49,964 (pooled) participants across the studies. Data collection began as early as 1946 and ended as recently as 2016. Most articles (n = 29) reported trajectories of more than one phenotype, but only five of these included phenotypes of more than one body system.

Most of the articles reported phenotype trajectories relevant to cardiovascular diseases (n = 19). These included trajectories of systolic blood pressure (n = 17), diastolic blood pressure (n = 15), heart rate (n = 1) and pulse wave velocity (n = 3). No eligible studies reported trajectories of retinal microvasculature vessel calibre, augmentation index or carotid artery intima media thickness. All five articles with trajectories underlying diabetes reported on glucose; none reported trajectories of HbA1c. One article reported trajectories of estimated glomerular filtration rate, relevant to chronic kidney disease, while none reported trajectories of albuminuria. One reported lung function trajectories, relevant to COPD, including forced expiratory volume in the first second (FEV_1_) and forced vital capacity (FVC). Five articles reported musculoskeletal phenotype trajectories, relevant to the adverse impacts of falls. These included trajectories of bone mineral content (n = 2), bone mineral density (n = 2), grip strength (n = 3) and muscle mass (n = 2). Articles (n = 10) reporting cognition trajectories (important to Alzheimer’s disease) spanned diverse cognitive phenotypes, tapping into areas including memory, processing speed, executive functioning, language and global cognition. We identified no articles reporting phenotype trajectories using longitudinal data for hearing or vision.

## Trajectory Mapping

### Individual Phenotype Synthesis

**Figure 2:**
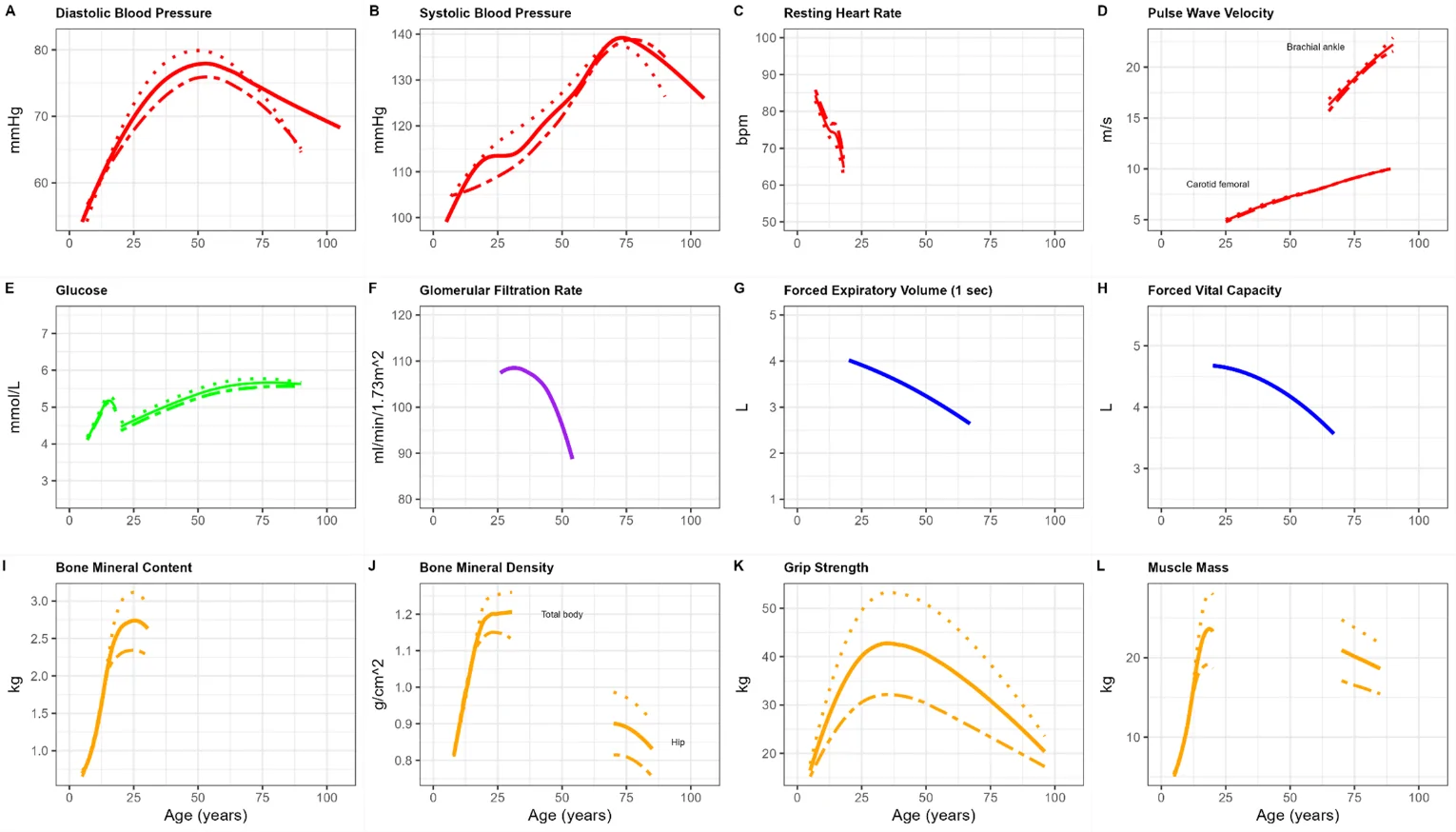
Figure 2: Synthesised Trajectories of Individual Phenotypes in Natural Units of Measurement

Figure 2 shows individual phenotype trajectories, each in their natural units of measurement, after synthesising across the included studies. As can be seen, the available longitudinal data for most phenotypes spanned only parts of the life course, with some limited to midlife and others to early and/or late life; blood pressure and grip strength were the notable exceptions with nearly whole-of-life data. We could not synthesise trajectories of cognition due to the diversity of measures used across the studies.

1. *Cardiovascular Disease:* The synthesised trajectories spanned ages 5-105 years for systolic and diastolic blood pressure; 7-18 years for heart rate; and 40-90 years for carotid-femoral and 65-90 years for brachial-ankle pulse wave velocity.
2. *Diabetes:* Synthesised trajectories of glucose spanned ages 7-18 years and 20-90 years.
3. *Chronic Kidney Disease:* Synthesised trajectories of glomerular filtration rate spanned ages 30-50 years.
4. *COPD:* Synthesised lung function trajectories spanned ages 20-68 years for both FEV_1_ and FVC.
5. *Adverse Impacts of Falls:* Collectively, synthesised trajectories of bone mineral content spanned ages 5-30 years, bone mineral density ages 8-30 years and 70-85 years, grip strength age 4-96 years and muscle mass ages 5-20 years and 70-85 years.
6. *Alzheimer’s Disease:* While trajectories of cognition could not be synthesised, they collectively spanned ages 43-105 years.

### Comparative Synthesis

**Figure 3:**
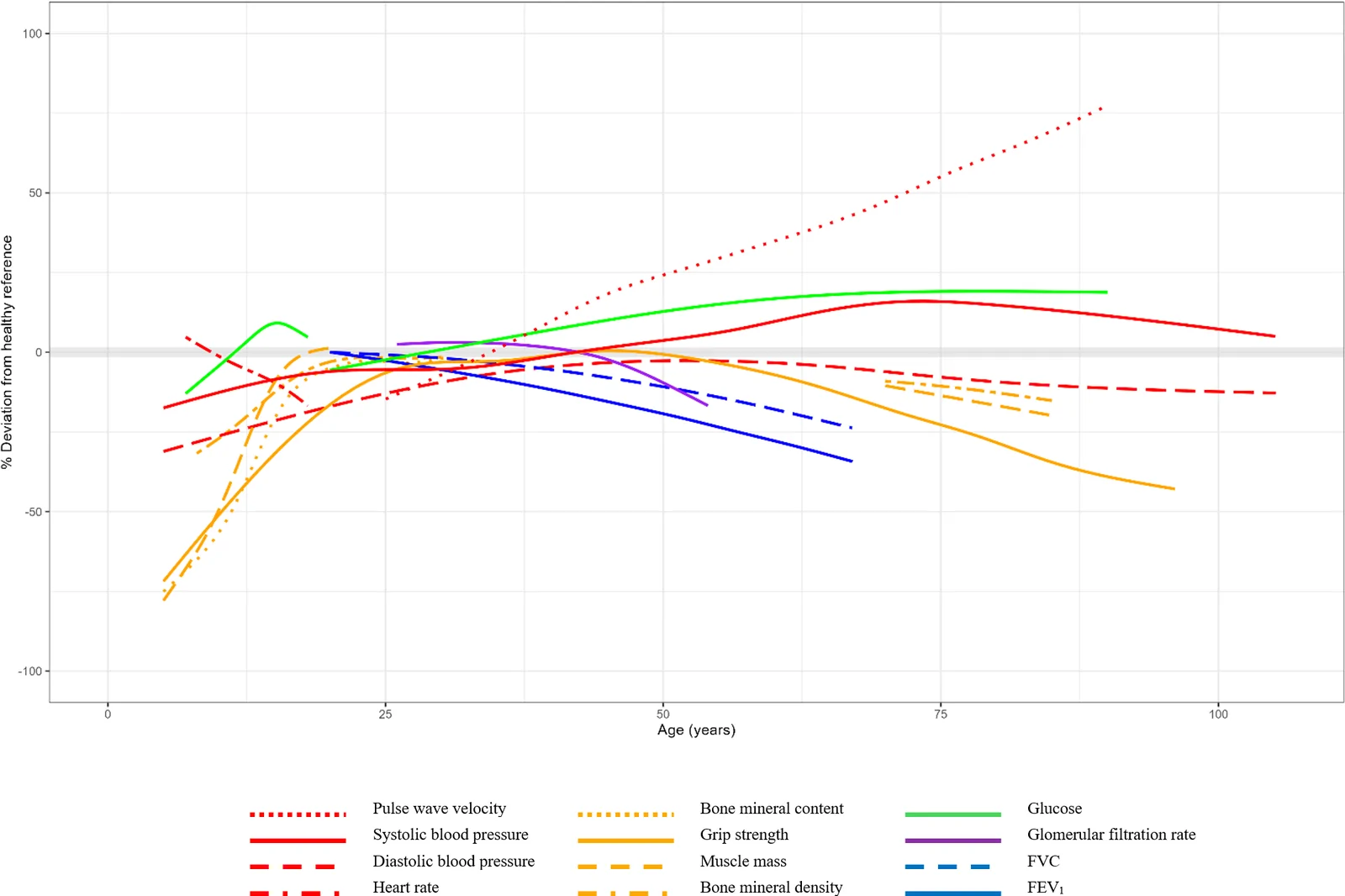
Figure 3: Comparative Synthesis of Phenotype Trajectories

Figure 3 shows synthesised trajectories of all phenotypes (except cognition) on a comparable scale, with the y-axis converted and standardised to a percentage deviation away from a healthy reference value for each phenotype. The musculoskeletal phenotypes follow a general pattern of childhood growth, early adult peak and decline with ageing. Bone mineral content and bone mineral density reach their peaks earlier than grip strength (occurring at approximately age 25, 30 and 35 years, respectively). Diastolic and systolic blood pressure, pulse wave velocity and glucose follow a general rising pattern across the life course. Glucose and pulse wave velocity begin to rise outside healthy ranges from early adulthood (at approximately age 30 and 32 years, respectively), whereas deviation above healthy range occurs later for systolic blood pressure (at approximately age 40 years). Diastolic blood pressure did not rise outside a healthy range (i.e., above 80mmHg) in these synthesised trajectories. Trajectories of heart rate, kidney function and lung function did not span enough of the life course to determine the general patterns they each follow.

**Table 2:** Characteristics of Included Studies

**Reference; Country of cohort**	**Sample description**	**Sample size; proportion female**	**Years of follow up**	**Phenotype(s) measured and method**	**Trajectory analysis methods**	**Trajectory age span (years)**
AlGhatrif et al. (2013); USA	Participants in the Baltimore Longitudinal Study of Ageing with no evidence of clinical atherosclerosis or aberrant pulse wave velocity	N = 775; 55%	1988-2013	Pulse wave velocity – carotid-femoral using different devices (transcutaneous Doppler probes, Complior SP device, SphygmoCor system)	Linear mixed effect models; separated by sex	40-90
Bonati et al. (2014); Italy	Participants in the Gubbio Population Study with at least one participating relative	N = 2620; NR	Three waves between 1983-2007	Systolic and diastolic BP – sphygmomanometer	Latent curve models	5-89
Cheng et al. (2012); USA	Participants in the Framingham Offspring Study, excluding those <25 or ≥75 years old at examination, prevalent myocardial infarction or heart failure, or with missing data on main variables	N = 4993 (final examination); 52%	Six waves between 1971-1998	Systolic and diastolic BP – methods not described	Multilevel modelling; separated by sex	25-70
Das et al. (2020); Australia	Participants in the Mater-University of Queensland Study of Pregnancy	N = 3793 (first follow up); NR	1981-2004	Systolic and diastolic BP – sphygmomanometer	Mean distribution	5-30
Davis et al. (2017); UK	Participants in the MRC National Survey of Health and Development with at least one cognitive assessment and complete data on covariates	N = 3185; 49%	Not specified – regular follow up since birth in 1946	Verbal memory – recall of a 15-item word learning task; Visual search speed – task requiring participants to cross out the letters P and W, randomly embedded within a grid of other letters in one minute	Linear mixed models; separated by sex	43-69
Dekkers et al. (2002); USA	Participants in an ongoing longitudinal study investigating cardiovascular risk factors in young people	N = 745; NR	Annual from 1989-1999	Systolic and diastolic BP – automated oscillatory system	Individual growth curve modelling within a multilevel framework; separated by sex and ethnicity (Black, White)	9-21
Dodds et al. (2014); UK	Participants of 12 general population cohort studies in Great Britain	N = 49,964; 53%	Varies by cohort – between 1990-2012	Grip strength – Hand dynamometer	Centiles using the Box-Cox Cole and Green (BCCG) distribution; separated by sex	4-90+
Dodge et al. (2014); USA	Participants from two epidemiological studies of dementia: the Monongahela Valley Independent Elders Study (MoVIES) and the Monongahela-Youghiogheny Healthy Aging Team study (MYHAT). Excluded if age-education-adjusted Mini-Mental State Examination score less than or equal to 21 at recruitment	N = 3626; NR	Not specified – MoVIES recruitment between 1987-1989, MYHAT recruitment between 2005-2007	Executive functioning – Trail Making Test B and letter fluency; Attention/psychomotor speed – Trail Making Test A; Language – verbal fluency for the category of animals	Mixed-effects models; separated by birth cohorts	65-98
Gerstorf et al. (2006); Germany	A subsample of participants from the Berlin Aging Study (BASE). Excluded participants below an age cohort-specific cutoff on a dementia screening tool	N = 368; NR	Five waves between 1990-2005	Episodic memory – Paired Associates and Memory for Text tests; Perceptual speed – Digit Letter and Identical Pictures; Fluency – Categories and Word Beginnings tests; Knowledge – Vocabulary and Spot a Word tests	Individual growth models; separated by sex	70-100
Golchert et al. (2019); Germany	Participants in the German Study on Ageing, Cognition and Dementia in Primary Care Patients (AgeCoDe), which continued as the Study on Needs, Health Service Use, Costs and Health-Related Quality of Life in a Large Sample of Oldest-Old Primary Care Patients (85+) (AgeQualiDe). Included if completed all follow-ups	N = 3254; 65%	Nine waves approximately every 1.5 years since recruitment in 2003-2004	Episodic memory – Word List Learning Test (immediate and delayed free recall)	Multilevel linear mixed-effects models with unconditional growth models; separated by sex	Not specified – 9 years of follow up from age 75+
Hulman et al. (2014); UK	Participants in the Whitehall II study	N = 10,308; NR	Nine waves between 1985-2009.	Systolic and diastolic BP – sphygmomanometer	Mixed-effects models with random intercepts; separated by sex	23-80
Ji et al. (2020); USA	Participants from four community-based cohorts (the Framingham Heart Study offspring cohort, the Atherosclerosis Risk in Communities (ARIC) Study, the Coronary Artery Risk Development in Young Adults (CARDIA) Study, and the Multi-Ethnic Study of Atherosclerosis (MESA)). Excluded if missing data for BP measures or antihypertensive medication	N = 32,833; NR	Not specified – varies by cohort	Systolic and diastolic BP – sphygmomanometer	Mixed effect regression models; separated by sex	20-80
Korologou-Linden et al. (2019); UK	Participants in the Avon Longitudinal Study of Parents and Children (ALSPAC)	Ranges from N = 699 to 6953; NR	Not specified – recruitment 1991-1992.	Systolic and diastolic BP – not specified; fasting blood glucose – not specified	Linear spline multilevel models	7-18
Lu et al. (2016); USA	Participants in the Fels Longitudinal Study with at least 3/15 possible total body BMC and BMD measurements	N = 655; 52%	DXA measurements began in 1989	Total body BMD and BMC - DXA	Mixed-effects models; separated by sex	8-30
Marcon et al. (2021); international	Healthy participants in an international general population cohort study (European Community Respiratory Health Survey)	N = 3216; NR	Three waves between 1991-2013	FEV_1_ and FVC – Spirometry	Gaussian outcome distribution and identity link function	20-68
McCormack et al. (2017); USA	Participants in the Bone Mineral Density in Childhood Study, a study of healthy children without medical conditions known to negatively impact bone health and with a height and body mass index within the 3^rd^ – 97^th^ percentiles at study entry	N = 2014; 51%	Annual since recruitment in 2002-2010	BMC and muscle mass – DXA scans	Growth modelling approach called superimposition by translation and rotation (SITAR); separated by sex and race (African American, Non-African American)	5-20
McFall et al. (2019); USA	Participants in the Victoria Longitudinal Study (VLS). Excluded if missing episodic memory data on all three waves, brain-related health conditions, Mini-Mental State Examination score < 24, recent head injury, moderate to very serious stroke	N = 882; NR	Not specified	Episodic memory – Word recall and Rey Auditory Verbal Learning Test	Overall latent growth change	55-105
Nahhas et al. (2010); USA	Participants in the Fels Longitudinal Study with at least one hand grip strength measurement	N = 1031; 52%	Hand grip measurements between 1985-2008	Grip strength - Hand Dynamometer	Bayesian longitudinal plateau model; separated by sex and birth cohort	18-96
O’Keeffe et al. (2018); UK	Participants in the Avon Longitudinal Study of Parents and Children (ALSPAC)	N = 8057 (BP); N = 5480 (glucose); NR	1991-2000	Systolic BP, diastolic BP and heart rate – sphygmomanometer; glucose – fasting blood sample	Linear spline multilevel models; separated by sex	7-18
Peralta et al. (2013); USA	Participants in the CARDIA Study with a visit at year 10 or later and at least one cystatin C measurement	N = 3334; NR	Approximately every 5 years between 1985-2006.	Estimated glomerular filtration rate – cystatin C-based	Linear mixed models; separated by race (Black, White)	30-50 (approximate)
Reas et al. (2017); USA	Participants of the Rancho Bernardo Study of Healthy Ageing with at least one cognitive function assessment and who supplied information on educational attainment	N = 2225; NR	Between 1972-2016	Global cognition – MMSE; executive function – Trail Making Test Part B; verbal semantic fluency – Category fluency; verbal episodic memory – Buschke-Fuld Selective Reminding test	Mixed effect regression models; separated by sex	50-90
Schievink et al. (2022); Netherlands	Participants in the Maastricht Aging Study (MAAS) who were cognitively healthy	N = 1823; NR	Not specified	Executive functioning – Concept Shifting Test; Verbal memory – delayed recall of the visual Verbal Learning Test; Information processing speed – Letter Digit Substitution Test	Random effects models; separated by incident CVD and no incident CVD	Not specified – 12 years of follow up
Scuteri et al. (2014); Italy	Participants in the Sardinia of the National Institute of Aging (SardiNIA) study	N = 4358; 58%	Not specified	Systolic and diastolic BP – mercury sphygmomanometer; Pulse wave velocity – carotid-femoral using nondirectional transcutaneous Doppler probes	Linear mixed-effects regression model; separated by sex	30-70
Shen et al. (2017); USA	Participants in the Bogalusa Heart Study with 4-15 serial BMI and BP measurements	N = 2732; NR	Not specified – began in 1973	Systolic and diastolic BP – Mercury sphygmomanometer	Nonlinear growth curve parameters estimated using a random-effects mixed model; separated by race (Black, White) and sex	Approximately 5-50
Tampubolon (2015); UK	Participants in the English Longitudinal Study of Ageing (ELSA) with complete information on covariates	N = 5931; NR	2002-2013	Episodic memory – sum of delayed and immediate recall	Maximum likelihood estimator of growth curve model; separated by birth cohort	50-90
Taniguchi et al. (2018); Japan	Participants in a longitudinal study of ageing and health based in Kusatsu Town, Gunma Prefecture	N = 1744; NR	Baseline between 2003-2015	Pulse wave velocity – brachial-ankle using an automatic waveform analyser; separated by sex	Linear mixed-effects model	65-90
Ulmer et al. (2007); Austria	Participants in the Vorarlberg Health Monitoring and Promotion Programme (a population-based risk factor surveillance program)	N = 181350; NR	Fasting glucose between 1989-2005	Systolic BP, diastolic BP and fasting blood glucose – methods not specified	General Estimating Equation population-averaged models; separated by sex and birth cohort	20-80
van der Willik et al. (2021); Netherlands	Participants in the Rotterdam Study, excluding those above age 90 or with a diagnosis of dementia, stroke or Parkinson’s disease	N = 9514; NR	Cognitive assessments between 1997-2016	Executive functioning, processing speed, recall and visuospatial abilities – neuropsychological battery including word fluency, letter digit substitution, stroop, word learning and design organisation tests	Linear mixed models	45-90
Vonk et al. (2019); USA	Participants in the Washington Heights Inwood Columbia Aging Project who were free of dementia	N = 1806 (most recent birth cohort included in this review, born 1921-1935); NR	Six visits over 17 years since recruitment in 1999.	Memory - total recall, delayed recall, and delayed recognition on the Selective Reminding Test; Language - 15-item Boston Naming Test, letter and category fluency, the Similarities subtest from the Wechsler Adult Intelligence Scale-Revised and the Repetition and Comprehension subtests of the Boston Diagnostic Aphasia Evaluation; Visuospatial performance - Recognition and Matching tasks on the Benton Visual Retention Test, the Rosen Drawing Test, and the Identities and Oddities subtest from the Mattis Dementia Rating Scale; Global cognition – average composite scores of other domains	Latent growth curve modelling	Not specified – 17 years of follow up
Wang et al. (2019); Sweden	Participants in the Swedish National study on Aging and Care in Kungsholmen (SNAC-K) with BP measurements at baseline	N = 3315; 65%	Every 3-6 years since recruitment in 2001-2004	Systolic and diastolic BP – Digital sphygmomanometer	Mixed-effects models; separated by birth cohort	60-100+
Westbury et al. (2020); USA	Participants in the Health ABC Study with data on at least one variable at two or more timepoints	N = 2917; 51%	Annually for 10 years since recruitment in 1997-1998.	Bone density – whole body DXA, muscle mass – whole body DXA, grip strength – Jamar dynamometer	Linear mixed effect models; separated by sex	70-85
Wills et al. (2011); UK	Participants with at least 2 repeat measurements of BP from 8 cohorts (Caerphilly Prospective Study, Hertfordshire Ageing Study, Medical Research Council National Survey of Health and Development, West of Scotland Twenty-07 study, Avon Longitudinal Study of Parents and Children, Whitehall II study)	N = 30,372; NR	Not described (varies by cohort)	Systolic BP - Sphygmomanometer	Multilevel models; separated by sex	7-80+
Wills et al. (2012); UK	Participants in the MRC National Survey of Health and Development with at least one BP measure	N = 3659; 50%		Systolic and diastolic BP – Sphygmomanometer	Median pattern (percentiles); separated by sex	36-53
Yaffe et al. (2021); USA	Participants from four cohort studies (Coronary Artery Risk Development in Young Adults study (CARDIA), Multi-Ethnic Study of Atherosclerosis (MESA), the Cardiovascular Health Study (CHS), and the Health, Aging and Body Composition study (Health ABC) of older adults) with at least two repeat measures of CVD risk factors	N = 15,001; NR	Not described (varies by cohort)	SBP – not described; fasting blood glucose – not described	Linear mixed models	20-90
Zeki Al Hazzouri et al. (2019); USA	Participants from four prospective cohort studies (CARDIA, MESA, CHS, Health ABC) with at least two repeated measurements of each CVD risk factor	N = 15,001 (pooled); NR	Varies by cohort – recruitment 1985-1986 in CARDIA, 2000-2001 in MESA, 1990 in CHS and 1997 in Health ABC	Systolic and diastolic BP –not specified; fasting blood glucose – not specified	Linear mixed models; separated by sex and race (Black, White)	20-90
Zhang et al. (2018); USA	Adult participants in the Bogalusa Heart Study with at least 4 BP and BMI measurements (with at least 2 in childhood)	N = 1154; NR	Between 1973-2010	Systolic and diastolic BP – Sphygmomanometer	Nonlinear growth curves estimated using random-effects mixed model; separated by sex and race (Black, White)	4-51

NR = not reported.

Trajectories were synthesised across studies by fitting loess curves through available data points from each included study. Separate trajectories for males and females were synthesised only where included studies separated trajectories by sex. Cognition trajectories could not be synthesised due to measurement diversity.

Trajectories were synthesised across studies by first converting available data points to a percentage deviation from a healthy reference value, then by fitting loess curves.

## Discussion

This review revealed stark gaps in the mapping of precursor phenotypes to major NCDs in high-income countries. While some phenotypic trajectories are well documented across multiple life stages, others remain incomplete. The extent and timing of these gaps varies by phenotype, with some missing coverage in midlife and others in childhood and/or older adulthood. We identified longitudinal trajectories of some, but not all, core phenotypes underlying cardiovascular disease, diabetes and adverse impacts of falls across the life course; for example, grip strength measures spanned most age ranges, but measures of bone mineral content did not. These findings highlight the need for more comprehensive, long-term tracking of phenotypic pathways to better inform NCD prevention and treatment strategies.

A strength of our synthesis method is that it enabled us to map trajectories of multiple phenotypes simultaneously in both absolute and relative terms and, where data were available, across the entire life course. This allowed us to take an interconnected approach to all major causes of disease burden. While other studies have assessed multisystem trajectories (e.g., Niiranen et al., 2019 [74]), we are not aware of any other publications that have attempted this for the entire life course, or that have standardised trajectories to enable comparison across phenotypes.

Our review also has limitations. The cohorts varied by ethnicity, birth year and socioeconomic factors. These features can impact trajectory patterns but were beyond the scope of this review to explore. Nonetheless, our synthesis process therefore risks mapping trajectories that no cohort actually experienced. Our synthesis did not account for factors like attrition, selection bias or treatment effects. Further limitations include not conducting formal quality appraisal or sensitivity analyses (e.g., to assess the impact of standardising to alternative healthy reference values) and imprecision from using PlotDigitizer to extract data and loess smoothing to interpolate data between data points. Our synthesis was intended to map available evidence and gaps to inform future data collection, and not to inform clinical decisions. Still, these methodological choices could have introduced bias and influenced the trajectory patterns we generated. Our focus on normative population trajectories led us to exclude alternative approaches, including latent class and cross-sectionally derived trajectories. These approaches provide insights into heterogeneity and life course patterning, and their exclusion may underestimate the broader evidence base. However, they do not produce a single, population-level trajectory that can prospectively inform universal measurement, timing and outcome selection within large cohort studies. Some apparent gap (e.g., lung or kidney function) may therefore reflect analytic choices rather than absence of underlying data, with opportunities for future synthesis and re-analysis [75]. To contain scope, this review focused on the highest-burden NCDs, excluding others whose importance may rise. Because we sought to inform planning for an Australian cohort, we excluded studies reporting population trajectories in LMICs. Given the growing burden from NCDs in LMICs, [76] it is important for future research to similarly map available evidence and gaps in these settings. Future work could also map other trajectories (such as lipids or phenotypes relevant to other NCDs) or those modelled using methods we excluded.

We can speculate as to why the gaps we demonstrate might exist, despite likely contributing to substantive NCD burden. Measurement expense, burden or invasiveness may explain some gaps, for example why in the cardiovascular system we identified substantial evidence for blood pressure trajectories but none of intima-media thickness. How health issues are framed can also shape their prioritisation in research [77]. Powerful actors including the World Health Organization and NCD Alliance have emphasised the threats from just five NCDs (cardiovascular diseases, cancer, diabetes, chronic respiratory diseases and mental health); while in no way downplaying their importance, this may influence cohorts to focus less on other major causes of disease burden [78].

If key phenotypes continue to be overlooked, our understanding of pathways to major NCDs will remain fragmented. Cohort studies are our primary avenue for capturing repeated population-level measurements and testing interventions during silent precursor phases. Systematic ‘black holes’ in measurement preclude them from informing public health and clinical priorities. A further challenge is the heterogeneity in life course cognitive metrics, reflecting lack of standardisation in the field [79]. While efforts to establish common batteries and core outcomes are underway [80], no consensus has emerged. This limits comparability across and continuity within cohorts, making it harder to identify and understand dementia progression or track the long-term impacts of cognitive interventions [81].

Our prior review [82] of NCD phenotypes measured in contemporary child cohorts identified many of the same measurement gaps now evident in their derived trajectories – meaning that, without action, these ‘missing’ trajectories will remain so. Addressing this requires cohorts to plan future data collection waves to fill gaps and map trajectories. This review informs GenV’s planning in four ways: (1) identifying priority gaps (e.g., hearing, vision) to be incorporated and repeated across 5-yearly phenomic waves to enable population scale trajectory mapping; (2) providing trajectory parameters for existing phenotypes to support power calculations and outcome selection for embedded trials; (3) assisting with timing of measurement and intervention evaluation based on observed trajectory shapes; and (4) guiding harmonisation with, and extension beyond, international cohorts. Together, these support a deliberate shift towards scalable, inclusive measures across cohorts, even where this trades off against gold-standard precision. Beyond these, neglected phenotypes and their related NCDs must be reframed to align their known impacts on morbidity and mortality with research priorities.

## Conclusion

Few phenotypes underlying major NCDs have well-mapped lifelong trajectories. Population-based cohorts could redress this by tracking key phenotypes over time, revealing long-term pathways to disease and enabling large-scale testing of real or virtual interventions. Until these gaps are filled, some of the largest contributors to global disease burden risk being overlooked in public health priorities.

## Data Availability

The data underlying this review article are available on reasonable request to the corresponding author (Prof Melissa Wake).
