# Supplementary material for: Gaps in Population Life Course Phenotype Trajectories Underlying Major Noncommunicable Diseases: A Scoping Review
Source: Int J Popul Data Sci. 2026 Aug 20;11(1):3346. doi: 10.23889/ijpds.v6i1.3346 (PMC13494735; doi:10.23889/ijpds.v6i1.3346)
Supplement: Supplementary file 1 [file ijpds-11-3346-s001.pdf]

Supplementary Material S1: Preferred Reporting Items for Systematic reviews and Meta-Analyses extension for Scoping Reviews (PRISMA-ScR) checklist

Section	Item	Prisma-ScR checklist item	Reported on page #
**Title**			
Title	1	Identify the report as a scoping review.	1
**Abstract**			
Structured summary	2	Provide a structured summary that includes (as applicable): background, objectives, eligibility criteria, sources of evidence, charting methods, results, and conclusions that relate to the review questions and objectives.	2
**Introduction**			
Rationale	3	Describe the rationale for the review in the context of what is already known. Explain why the review questions/objectives lend themselves to a scoping review approach.	3
Objectives	4	Provide an explicit statement of the questions and objectives being addressed with reference to their key elements (e.g., population or participants, concepts, and context) or other relevant key elements used to conceptualise the review questions and/or objectives.	3
**Methods**			
Protocol and registration	5	Indicate whether a review protocol exists; state if and where it can be accessed (e.g., a Web address); and if available, provide registration information, including the registration number.	4
Eligibility criteria	6	Specify characteristics of the sources of evidence used as eligibility criteria (e.g., years considered, language, and publication status), and provide a rationale.	4-5
Information sources*	7	Describe all information sources in the search (e.g., databases with dates of coverage and contact with authors to identify additional sources), as well as the date the most recent search was executed.	4
Search	8	Present the full electronic search strategy for at least 1 database, including any limits used, such that it could be repeated.	Supplementary material S2
Selection of sources of evidence†	9	State the process for selecting sources of evidence (i.e., screening and eligibility) included in the scoping review.	5
Data charting process‡	10	Describe the methods of charting data from the included sources of evidence (e.g., calibrated forms or forms that have been tested by the team before their use, and whether data charting was done independently or in duplicate) and any processes for obtaining and confirming data from investigators.	5
Data items	11	List and define all variables for which data were sought and any assumptions and simplifications made.	5
Critical appraisal of individual sources of evidence§	12	If done, provide a rationale for conducting a critical appraisal of included sources of evidence; describe the methods used and how this information was used in any data synthesis (if appropriate).	NA
Synthesis of results	13	Describe the methods of handling and summarising the data that were charted.	5
**Results**			
Selection of sources of evidence	14	Give numbers of sources of evidence screened, assessed for eligibility, and included in the review, with reasons for exclusions at each stage, ideally using a flow diagram.	6
Characteristics of sources of evidence	15	For each source of evidence, present characteristics for which data were charted and provide the citations.	6-7
Critical appraisal within sources of evidence	16	If done, present data on critical appraisal of included sources of evidence (see item 12).	NA

Continued

Supplementary Material S1: Continued

**Section**	**Item**	**Prisma-ScR checklist item**	**Reported on page #**
Results of individual sources of evidence	17	For each included source of evidence, present the relevant data that were charted that relate to the review questions and objectives.	7
Synthesis of results	18	Summarise and/or present the charting results as they relate to the review questions and objectives.	16-17
**Discussion**			
Summary of evidence	19	Summarise the main results (including an overview of concepts, themes, and types of evidence available), link to the review questions and objectives, and consider the relevance to key groups.	18
Limitations	20	Discuss the limitations of the scoping review process.	19
Conclusions	21	Provide a general interpretation of the results with respect to the review questions and objectives, as well as potential implications and/or next steps.	19
**Funding**			
Funding	22	Describe sources of funding for the included sources of evidence, as well as sources of funding for the scoping review. Describe the role of the funders of the scoping review.	20

JBI = Joanna Briggs Institute; PRISMA-ScR = Preferred Reporting Items for Systematic reviews and Meta-Analyses extension for Scoping Reviews.

*Where *sources of evidence* (see second footnote) are compiled from, such as bibliographic databases, social media platforms, and Web sites.

† A more inclusive/heterogeneous term used to account for the different types of evidence or data sources (e.g., quantitative and/or qualitative research, expert opinion, and policy documents) that may be eligible in a scoping review as opposed to only studies. This is not to be confused with *information sources* (see first footnote).

‡ The frameworks by Arksey and O'Malley (6) and Levac and colleagues (7) and the JBI guidance (4, 5) refer to the process of data extraction in a scoping review as data charting.

§The process of systematically examining research evidence to assess its validity, results, and relevance before using it to inform a decision. This term is used for items 12 and 19 instead of "risk of bias" (which is more applicable to systematic reviews of interventions) to include and acknowledge the various sources of evidence that may be used in a scoping review (e.g., quantitative and/or qualitative research, expert opinion, and policy document).

From: Tricco AC, Lillie E, Zarin W, O'Brien KK, Colquhoun H, Levac D, et al. PRISMA Extension for Scoping Reviews (PRISMAScR): Checklist and xplanation. Ann Intern Med. 2018;169:467–473. https://doi.org/10.7326/M18-0850.

Supplementary material S2

Ovid MEDLINE search strategy
1 cardiovascular diseases/ or exp cardiovascular abnormalities/ or exp heart diseases/ or exp vascular diseases/
2 exp asthma/ or exp pulmonary disease, chronic obstructive/
3 diabetes mellitus/ or exp diabetes mellitus, type 1/ or exp diabetes mellitus, type 2/ or latent autoimmune diabetes in adults/ or prediabetic state/
4 exp Osteoporosis/
5 Accidental Falls/
6 Sarcopenia/
7 exp kidney diseases/ and chronic.tw,kf,hw.
8 Presbycusis/
9 exp Hearing Loss/
10 exp Blindness/
11 Alzheimer Disease/
12 (Chronic-Obstructive-Pulmonary-Disease* or COPD or Cardiovascular-Disease* or Heart-Disease* or stroke or Diabetes or Osteoporosis or accidental-fall* or Sarcopenia* or Kidney-Disease* or Presbycusis or hearing or deaf* or blind* or vision or Alzheimer* or dementia*).tw,kf.
13 1 or 2 or 3 or 4 or 5 or 6 or 7 or 8 or 9 or 10 or 11 or 12
14 (ph or ep or et or pp).fs.
15 exp cohort studies/
16 (longterm or long-term or repeat* or serial or longitudinal* or follow-up or followup or cohort* or retrospective* or prospective*).tw,kf.
17 15 or 16
18 (life-course* or trajector*).tw,kf.
19 *Blood Pressure/
20 exp *forced expiratory flow rates/ or *forced expiratory volume/
21 exp *Muscle Strength/
22 exp *Vital Capacity/
23 *respiratory function tests/ or *airway resistance/ or *lung compliance/ or exp *lung volume measurements/ or exp *spirometry/
24 *carotid intima-media thickness/ or *vascular stiffness/
25 exp *Pulse Wave Analysis/
26 exp *Retinal Vessels/
27 *Blood Glucose/
28 *Glycated Hemoglobin A/
29 exp *bone density/ or exp *muscle strength/ or exp *hand strength/ or exp *musculoskeletal development/
30 *Albuminuria/
31 exp *Glomerular Filtration Rate/
32 exp *Auditory Threshold/
33 exp *Visual Acuity/
34 *cognition/ or exp *cognitive reserve/ or *executive function/
35 (blood-pressure or vascular-function* or vascular-stiffness or augmentation-index or pulse-pressure or pulse-wave-velocit* or retinal-microvasculature* or carotid-artery-intima-media-thickness or cIMT or carotid-artery-distensibilit* or carotid-artery-elasticit* or lung-function* or FEV or FVC or respiratory-function* or forced-expiratory-flow-rate* or forced-expiratory-volume* or spirometry vital-capacit* or respiratory-function-test* or lung-volume-measurement* or blood-glucose or HbA1c or bone-densit* or bone-morpholog* or bone-strength or bone-geometr* or muscle-strength or muscle-densit* or muscle-morpholog* or grip-strength or hand-strength or albuminuria or glomerular-filtration-rate* or vision or cognition or cognitive-reserve or verbal-iq or performance-iq or executive-function*).tw,kf.
36 19 or 20 or 21 or 22 or 23 or 24 or 25 or 26 or 27 or 28 or 29 or 30 or 31 or 32 or 33 or 34 or 35
37 13 and 17 and 18 and 36
38 14 and 17 and 18 and 36
39 37 or 38
40 limit 39 to (case reports or comment or editorial or guideline or letter or practice guideline or preprint)
41 39 not 40

Supplementary Material S3: Healthy reference values used to standardise phenotype trajectories

Phenotype	Reference value	Sources of evidence
Systolic blood pressure	120 mmHg	Whelton, P. K., Carey, R. M., Aronow, W. S., Casey, D. E., Jr, Collins, K. J., Dennison Himmelfarb, C., DePalma, S. M., Gidding, S., Jamerson, K. A., Jones, D. W., MacLaughlin, E. J., Muntner, P., Ovbiagele, B., Smith, S. C., Jr, Spencer, C. C., Stafford, R. S., Taler, S. J., Thomas, R. J., Williams, K. A., Sr, Williamson, J. D., . . . Wright, J. T., Jr (2018). 2017 ACC/AHA/AAPA/ABC/ACPM/AGS/APhA/ASH/ASPC/NMA/PCNA Guideline for the Prevention, Detection, Evaluation, and Management of High Blood Pressure in Adults: Executive Summary: A Report of the American College of Cardiology/American Heart Association Task Force on Clinical Practice Guidelines. *Hypertension (Dallas, Tex. : 1979)*, *71*(6), 1269–1324. https://doi.org/10.1161/HYP.0000000000000066
Diastolic blood pressure	80 mmHg	Whelton, P. K., Carey, R. M., Aronow, W. S., Casey, D. E., Jr, Collins, K. J., Dennison Himmelfarb, C., DePalma, S. M., Gidding, S., Jamerson, K. A., Jones, D. W., MacLaughlin, E. J., Muntner, P., Ovbiagele, B., Smith, S. C., Jr, Spencer, C. C., Stafford, R. S., Taler, S. J., Thomas, R. J., Williams, K. A., Sr, Williamson, J. D., . . . Wright, J. T., Jr (2018). 2017 ACC/AHA/AAPA/ABC/ACPM/AGS/APhA/ASH/ASPC/NMA/PCNA Guideline for the Prevention, Detection, Evaluation, and Management of High Blood Pressure in Adults: Executive Summary: A Report of the American College of Cardiology/American Heart Association Task Force on Clinical Practice Guidelines. *Hypertension (Dallas, Tex. : 1979)*, *71*(6), 1269–1324. https://doi.org/10.1161/HYP.0000000000000066
Heart rate	80 beats per minute	Mason, J.W., Ramseth, D.J., Chanter, D.O., et al. Electrocardiographic reference ranges derived from 79,743 ambulatory subjects. *Journal of Electrocardiology*. 2007 Jul; *40*(3):228-234. DOI: 10.1016/j.jelectrocard.2006.09.003
Pulse wave velocity (carotid-femoral)	5.86 metres per second	Díaz, A., Galli, C., Tringler, M., Ramírez, A., & Cabrera Fischer, E. I. (2014). Reference values of pulse wave velocity in healthy people from an urban and rural argentinean population. *International journal of hypertension*, *2014*, 653239. https://doi.org/10.1155/2014/653239
Pulse wave velocity (brachial-ankle)	12.3 metres per second	Yiming, G., Zhou, X., Lv, W., Peng, Y., Zhang, W., Cheng, X., Li, Y., Xing, Q., Zhang, J., Zhou, Q., Zhang, L., Lu, Y., Wang, H., & Tang, B. (2017). Reference values of brachial-ankle pulse wave velocity according to age and blood pressure in a central Asia population. *PloS one*, *12*(4), e0171737. https://doi.org/10.1371/journal.pone.0171737
Glucose	4.75 mmol/L	World Health Organization. (2024, May 24). *Mean fasting blood glucose.* https://www.who.int/data/gho/indicator-metadata-registry/imr-details/2380
Glomerular filtration rate	105 mL/min/1.73m^2	National Kidney Foundation. (2023, Sep 05). *Understanding your lab values and other CKD health numbers.* https://www.kidney.org/kidney-topics/understanding-your-lab-values-and-other-ckd-health-numbers
FEV_1	Trajectory peak	Not applicable – peak available.
FVC	Trajectory peak	Not applicable – peak available.
Bone mineral content	Trajectory peak	Not applicable – peak available.
Bone mineral density (whole body)	Trajectory peak	Not applicable – peak available.
Bone mineral density (hip)	1.04 g/cm^2 (male) and 0.942 g/cm^2 (female)	Looker, A. C., Wahner, H. W., Dunn, W. L., Calvo, M. S., Harris, T. B., Heyse, S. P., Johnston, C. C., Jr, & Lindsay, R. (1998). Updated data on proximal femur bone mineral levels of US adults. *Osteoporosis International : a journal established as result of cooperation between the European Foundation for Osteoporosis and the National Osteoporosis Foundation of the USA*, *8*(5), 468–489. https://doi.org/10.1007/s001980050093

Continued

Supplementary Material S3: Continued

**Phenotype**	**Reference value**	**Sources of evidence**
Grip strength	Trajectory peak	Not applicable – peak available.
Muscle mass	28.16 kg (male) and 18.72 kg (female)	Gould, H., Brennan, S. L., Kotowicz, M. A., Nicholson, G. C., & Pasco, J. A. (2014). Total and appendicular lean mass reference ranges for Australian men and women: the Geelong osteoporosis study. *Calcified tissue international*, *94*(4), 363–372. https://doi.org/10.1007/s00223-013-9830-7
